# Change in brain activation after transcranial pulsed electromagnetic fields in treatment-resistant depression

**DOI:** 10.1007/s00406-024-01797-w

**Published:** 2024-04-05

**Authors:** Sjoerd M. van Belkum, Esther M. Opmeer, Hanneke Geugies, Marrit K. de Boer, Robert A. Schoevers, André Aleman

**Affiliations:** 1 Department of Psychiatry, Research School of Behavioral and Cognitive Neurosciences (BCN), Interdisciplinary Center Psychopathology of Emotion Regulation (ICPE), University Medical Center Groningen, University of Groningen, PO Box 30.001 (CC30), 9700 RB Groningen, The Netherlands; 2 Department of Neuroscience, University Medical Center Groningen, University of Groningen, Groningen, The Netherlands

**Keywords:** Depression, MDD, TMS, tPEMF, fMRI, Reward-processing

## Abstract

**Background:**

Preliminary evidence suggests antidepressant effects of transcranial pulsed electromagnetic fields (tPEMF). However, the precise mechanism of action in the brain is still unknown. The aim of this study was to investigate the influence of tPEMF on brain activation in patients with treatment-resistant depression (TRD) by studying two processes that might be of particular interest in relation to the symptoms of depression: emotional processing and reward processing.

**Methods:**

Eligible participants (*n* = 50) with TRD in this sham-controlled double-blind multicenter trial [registered at the Dutch Trial Register (http://www.trialregister.nl), NTR3702] were randomly assigned to five weeks daily active or sham tPEMF. Pre- and post-treatment functional MR-scans were made during which participants performed a social-emotional task and a reward task.

**Results:**

Participants in the active treatment group showed a stronger decrease in activation post-treatment compared to sham during reward-outcome processing in the left inferior frontal gyrus and in a cluster comprising the right lingual gyrus and the posterior part of the middle temporal gyrus. No effect of tPEMF was found on activation during the social-emotional task. Neurostimulation with tPEMF did also not affect behavioral performance for both tasks.

**Conclusions:**

We found a decrease in reward-related activation as a result of tPEMF stimulation, while no effect of tPEMF on social-emotional processing was found. The treatment-related reduction in activation of regulatory regions may reflect normalization and may have implications for anhedonia. These findings suggest that there is an effect of tPEMF on brain activation of relevant circuits, albeit in the absence of a clinical antidepressant effect.

## Introduction

Non-invasive brain stimulation is a promising new treatment approach for Major Depressive Disorder (MDD). Repetitive Transcranial Magnetic Stimulation (rTMS) [1, 2], a stimulation technique aimed at the activation of local brain regions through depolarization of the neuronal membrane, has shown consistent antidepressant effects [3]. Global stimulation with weak electromagnetic fields may also have beneficial effects, with simultaneous stimulation at multiple scalp sites and without depolarization [4, 5]. This technique uses smaller devices and without the use of a localization paradigm. An example is stimulation with transcranial Pulsed Electromagnetic Fields (tPEMF) [6], which at this point has yielded mixed results. One RCT has shown a positive effect of tPEMF on depression severity in MDD patients [6]. In a recent study using a similar setup but utilizing a much lower magnetic field strength, we found no antidepressant effect [7]. There is thus some preliminary evidence of the antidepressant effects of tPEMF, although the precise mechanism of action on the brain is still unclear [5]. Therefore, as an integral part of this latter RCT we evaluated the effects of tPEMF on brain activation.

To investigate the influence of tPEMF on the brain of patients with treatment-resistant depression (TRD), two processes might be of particular interest: emotional processing and reward processing. MDD has been associated with a change in emotional cognitive processing, in particular a biased interpretation of negative information [8–10], which manifests in the perception and identification of facial emotions [11, 12], and during emotional attention tasks [10]. One task combining this is the Wall of Faces (WoF), an emotional attention task [13]. In healthy participants increased activation was found in the anterior cingulate cortex (ACC) and ventral medial prefrontal cortex (VMPFC) among others during emotional trials compared to control trials [13]. These particular areas have also been found to be more activated during emotional processing in depressed patients, although a larger network of also limbic areas seems to be involved [14, 15]. Stimulation with rTMS on the left dorsolateral PFC (DLPFC) has been found to indirectly decrease activation in the ACC and VMPFC [16]. It could be hypothesized that as a result of tPEMF stimulation, changes could occur in activation in the areas involved in emotional processing.

Another important mechanism underlying core symptoms of MDD, with anhedonia in particular, is reward processing [17]. The reward system is driven by a frontostriatal circuit comprising the ACC, the orbital PFC, the (ventral and dorsal) striatum, the ventral pallidum, and the midbrain dopamine neurons [18]. During a monetary incentive delay (MID) task, it has been shown that patients with MDD show hyporesponsivity of the left caudate and hyperresponsivity of the bilateral middle frontal gyrus, the right ACC, and right orbital frontal lobe during reward anticipation, and hyporesponsivity of the left caudate during reward consumption [19]. This activity may change after treatment for depression, in structures that mediate responses to rewards, including the right caudate nucleus during reward anticipation, and the paracingulate and orbital frontal gyri during reward feedback [20]. In preclinical in vitro studies it has been shown that PEMF signals influence rodent dopaminergic cells [21]. Therefore, it could be hypothesized that by targeting the dopamine system, tPEMF may influence reward processing in this frontostriatal circuit.

The aim of this study was to investigate the influence of tPEMF on the brain activation of patients with TRD. Therefore, patients performed an emotion attention task and a reward-processing task during fMRI.

## Methods

### Study design

We included 55 depressed patients in a double-blind, multicenter RCT comparing active tPEMF versus sham in a 1:1 ratio. Fifty completed the trial and were included in the current analyses. This study was approved by the Medical Ethical Committee of the University Medical Center Groningen (UMCG), and by the local research office of each participating site. Written informed consent was obtained from each participant. The study was conducted according to the Declaration of Helsinki. The trial was registered at the Dutch Trial Register (http://www.trialregister.nl) under number NTR3702.

### Study population

We included patients who met DSM-IV criteria for MDD, currently in a depressive episode, assessed by the Mini-International Neuropsychiatric Interview (MINI) [22]. Inclusion criteria were the presence of at least a moderately severe depression (> 17 on HAMD-17), non-responsiveness to one or more antidepressants given for at least 4 weeks in an adequate dose (i.e. the defined daily dose (DDD) [23]) during the current episode, age between 18 and 80 years, and having a good understanding of spoken and written Dutch.

Patients were excluded if they were Magnetic Resonance Imaging (MRI) incompatible (having metal implants, claustrophobia, refusal to get informed of structural brain abnormalities, and suspected pregnancy). Additional exclusion criteria were presence of MDD with psychotic features, other major psychiatric disorders such as a primary psychotic disorder or an antisocial or borderline personality disorder, a neurological disorder such as dementia or epilepsy, visual or hearing problems that could not be corrected, suicidal thoughts (> 2 on HAMD-17 for suicidal ideation) or a history of a serious suicide attempt, recent (past three months) alcohol or drug abuse or dependence, lactation, inability to comply with treatments and/or assessments, recent change (last 4 weeks) in antidepressant medication or requirement to change antidepressant medication during the course of the study, use of benzodiazepine(s) more than 2 mg lorazepam or equivalent per day within the last 4 weeks or during the course of the study, use of medication indicated for a somatic disease that may have affected mood within the last 4 weeks, excessive use of coffee (> 10 units per day) or alcohol (> 5 units per day), or recent use (within four weeks) of cannabis or any other non-prescribed psychotropic drugs or unwillingness to abstain from these substances during the study. The use of antipsychotics and lithium was allowed.

### Treatment

Eligible patients were randomly assigned to either 5 weeks active tPEMF or 5 weeks sham stimulation. Sessions took place on weekdays for 30 min during office hours. All involved were blinded for the treatment condition. Further details regarding randomization and treatment procedure are described elsewhere [7]. Change in depression severity was measured by the HAMD-17 [24] immediately post-treatment. Functional MR scans were made at a maximum of five days pre-treatment and on the same day of the last treatment session. During the scans, participants performed two tasks: the Wall-of-Faces (WoF) task [13] and a Monetary Incentive Delay (MID) task [25].

### WoF-Task

During the WoF task, an array of 32 human faces was presented for 3 s followed by an additional reaction period of 1.5 s. Participants had to indicate whether they saw more happy or angry facial expressions –an affective judgment– or whether they saw more male or female faces—a gender judgment-, used as a control condition. In half of the trials, the majority of faces was clear (unambiguous trials) because the array was presented in a 26-6 or 6-26 ratio. In the other half of the trials the ratio of faces was presented in a 16-16 ratio and thus ambiguous. This resulted in two different ambiguous conditions (affect: angry = happy (16-16); gender: male = female (16-16)) and two unambiguous conditions (affect: angry ≠ happy (26-6 or 6-26); gender: male ≠ female (26-6 or 6-26)). In total, there were eight epochs; four in which participants had to perform an affective judgment and four in which they had to perform a gender judgment. During one epoch the ambiguous (four per epoch) and the unambiguous trials (four per epoch) were presented in random order. The four conditions were thus presented sixteen times in total. Stimuli were presented in E-prime version 2.0 (Psychology Software Tools, Sharpsburg, PA). A short practice session was performed prior to scanning.

### MID-task

The Monetary Incentive Delay task was adapted from [25]. This event-related task consisted of 20 reward trials (monetary gain), 20 neutral trials (no gain no loss), and twelve loss trials (monetary loss). The total reward obtained during scanning was added to the financial compensation for participation; the total amount was fixed so participants unknowingly always gained € 10,-. During a trial, participants saw a cue for 1.5 s for one of the potential outcomes: reward (+ €), neutral (= €), or loss (-€), which was followed by a blue squared target presented for 0.5 s. Participants were instructed to press a button in response to the cue as fast as possible to maximize their outcome. Feedback (1.5 s) concerning the outcome was given directly after the cue. The inter-stimulus-interval (ISI) varied between trials (ISI-1 between cue of the possible outcome and target: 3.5 s–9.5 s; ISI-2 between target and outcome: 2.5–8.5 s) to prevent expectancy effects, as did the duration of trial-separating fixation cross (3.0–7.0 s). Participants completed four blocks of thirteen trials comprising all conditions, interspersed with 10 s resting periods. The pseudo-randomized order of trials and ISIs was determined with optimized experimental design to maximize efficiency [26, 27] Reward success rates were set at 80% to prevent habituation. Outcome for the neutral and loss trials was set at 100%, so that in the neutral trials participants never received a reward and in the loss trials participants always lost money. Stimuli were presented in E-prime version 2.0 (Psychology Software Tools, Sharpsburg, PA). A short practice session was performed prior to scanning.

### MRI acquisition parameters

All fMRI-images were acquired using a 3 Tesla Philips MRI scanner (Best, The Netherlands). Functional images were acquired using T2*-weighted echo planar images sequences. Sequence parameters: single shot EPI; 37 slices; 3.5 mm slice thickness; 0.0 mm gap; 224 × 129.5 × 224 mm (anterior–posterior, foot-head, right-left) field of view; 64 × 61 scan matrix; transverse slice orientation; repetition time 2000 ms; echo time 20 ms; flip angle 70°. In addition, a T1-weighted whole-brain anatomical image was acquired (resolution 1 × 1 × 1 mm).

### Statistical analyses of behavioral data

Analyses of behavioral data were performed using IBM SPSS version 24.0 software (IBM, Chicago IL, USA). For the WoF-task we calculated median scores (non-normally distributed) for the response times of the conditions affect ambiguous, affect unambiguous, gender ambiguous, and gender unambiguous. For the MID-task, we calculated median scores for the response time to the target cue for the three conditions (reward, neutral, or loss). Outliers were calculated using the median absolute deviation (MAD) [28]. We used a criterion of 3 + or – the MAD for the different conditions to remove outliers. Differences between the conditions were tested using Wilcoxon rank tests for the median scores. To test for the effect of treatment on behavioral outcome, a linear mixed model with a random intercept was applied with response time score per different condition as dependent variable and treatment group, time (baseline or week 5), and the interaction between time and treatment group as covariates. The level of statistical significance was set at α < 0.05.

### MRI data pre-processing

Analyses of MRI data were performed using Statistical Parametric Mapping (SPM12, version number 6470; FIL Wellcome Department of Imaging Neuroscience, London, UK), implemented in MatLab (r2015a). First, PAR files were converted to NIFTI format with an in-house script. Both anatomical and functional images were manually reoriented to the anterior commissure – posterior commissure plane. Further preprocessing consisted of realignment of functional images. Realignment-parameters were visually checked. For all realignment-parameters, their first derivatives and the framewise displacement [29, 30] were calculated to add as covariate in the first-level model later on. Participants were excluded for that particular task if there was progressive movement exceeding 3 mm; in case a single volume would exceed 3 mm, we assumed that scrubbing would compensate for this. Next, coregistration of the functional images to the anatomical image, and spatial normalization to the Montreal Neurological Institute (MNI) space, reslicing the images into a 3 × 3 × 3 mm voxel grid were performed. Coregistration and normalization were visually checked to see if manual correction was necessary, which was not the case. The data was spatially smoothed with an 8 mm full-width at half-maximum Gaussian Kernel.

### First level models

For both tasks, a first-level GLM was set up per participant containing two time sessions. Regressors for the different onset times of the conditions were convolved with a canonical hemodynamic response function. Other regressors in the GLM were realignment parameters, their first derivatives, and dummy variables for the volumes showing a framewise displacement of > 0.9 [29, 30].

### Wall of faces task

For the WoF-task five different regressors were defined: affective ambiguous and unambiguous, gender ambiguous and unambiguous, and instructions. We calculated the following contrasts on baseline: affect > gender, ambiguous affect > ambiguous gender, affect ambiguity > affect unambiguity. For the interaction over time, we calculated contrasts for: affect > gender (pre > post), ambiguous affect > gender (pre > post), and affect ambiguity > unambiguity (pre > post),

### Monetary incentive delay task

For the MID-task six regressors were defined: anticipation of reward, loss, and neutral and consummation of reward, loss, and neutral. We calculated contrasts (at baseline) for anticipation reward > neutral, anticipation loss > neutral, anticipation reward > loss, consumption reward > neutral, consumption loss > neutral, and consumption reward > loss. For the effects over time, we calculated contrasts for anticipation reward > neutral (pre > post), anticipation loss > neutral (pre > post), anticipation reward > loss (pre > post), consumption reward > neutral (pre > post), consumption loss > neutral (pre > post), and consumption reward > loss (pre > post),

### Second level

For second-level analyses, a two-sample t-test model was built. We determined task activation using the baseline scans and compared baseline activation between the treatment groups (sham versus active). For the effect over time, we used the respective first-level contrasts to compare the treatment groups. We performed whole-brain correction. The threshold was set at *p* < 0.05 Family Wise Error (few)-corrected at the cluster level, with an initial voxel-defining threshold of *p* < 0.001.

To relate clinical findings to the outcome of the second-level analyses, we extracted the average effect size of the interaction for the Region Of Interest (ROI) defined by the respective clusters. Using regression analyses we explored the correlation between the ROI effect size and changes in HAMD-17-scores.

For visualization purposes, we built a full factorial model containing scans for the two groups and two time moments (pre-treatment, post-treatment for sham and active treatment). We used the significant clusters of the effect over time as a mask to extract the first eigenvariate for each condition and time moment and these were used for plotting bar graphs. No statistics were applied to these values.

## Results

### Sample description and clinical effects

Of the 50 participants who were included in the fMRI-analysis, 25 were randomized to the active and sham conditions. For the WoF-task five participants were excluded due to excessive movement, so we retained 45 participants for analysis of the WoF-task, 23 in the active and 22 in the sham group. For the MID-task, one participant was excluded due to excessive movement, retaining 49 participants for analysis: 25 in the active and 24 in the sham group. Table 1 shows that both treatment groups were comparable on socio-demographic data and clinical measurements.

**Table 1:** Sociodemographic and clinical parameters

Socio-demographics	Active		Sham		*P*-value	Test
*N*	25		25		–	
Age [years; mean (SD)]	49	14	45	12	0.331	2-tailed t-test
Gender (female %)	14	56%	11	44%	0.396	Chi-square
Marital status					0.768	Chi-square
Single (*n*; %)	10	40%	9	36%	–	
Married (*n*; %)	13	52%	15	60%	–	
Divorced (*n*; %)	2	8%	1	4%	–	
Educational background					0.321	Chi-square
Primary (*n*; %)	2	8%	0	0%	–	
Lower secondary (*n*; %)	6	24%	10	40%	–	
Upper secondary (*n*; %)	12	48%	12	48%	–	
University (*n*; %)	5	20%	3	12%	–	
Presence of somatic complaints (% yes)	16	64%	20	80%	0.208	Chi-square
MDD-type					0.395	Chi-square
MDD first episode (*n*; %)	12	48%	15	60%	–	
MDD recurrent episode (*n*; %)	13	52%	10	40%	–	
Number of episodes [median (IQR)]	1	(1–3)	1	(1–3)	0.782	Mann–Whitney U
Duration of current episode [mos; median (IQR)]	23	(13–54)	29	(12–78)	0.534	Mann–Whitney U
Presence of comorbidity (% yes)	14	56%	10	40%	0.258	Chi-square

*IQR* inter quartile range

For the clinical study, data were analyzed using the intent-to-treat (ITT) principle so that all 55 randomized participants were included in the analyses. We included 29 in the active treatment group and 26 in the sham treatment group. We observed an improvement in depression severity over time that continued for 15 weeks after the last stimulation (i.e., the last timepoint). However, we found no differences in improvement between the active treatment group and the sham group. Further details regarding the clinical effects are published elsewhere [7].

### Behavioral results—wall of faces task

There was no significant effect of group, no significant effect of time, and no significant group*time interaction for the affect ambiguous compared to the affect unambiguous condition, for all conditions.

### Behavioral results—monetary incentive delay task

There was no significant effect of group, no significant effect of time, and no significant group*time interaction for all conditions.

### Functional neuroimaging results—wall of faces task

The contrast affect > gender showed activation in the bilateral middle temporal gyrus (MTG, Table 2). For the contrast ambiguous affect > gender activation was found in the left lingual gyrus, left precentral gyrus, bilateral MTG, and right inferior frontal gyrus (IFG). For the affect ambiguity > affect unambiguity a difference in activation was seen in the right lingual gyrus.

**Table 2:** Functional neuroimaging results—wall of faces task

Area	*K*	*x*	*y*	*z*	*t*-score	*p*-value
Contrast affect vs. gender						
Middle temporal gyrus (right)	378	54	– 34	– 4	5.57	< 0.001
Middle temporal gyrus (left)	352	– 60	– 37	2	5.30	< 0.001
Contrast ambiguous affect vs. gender						
Lingual gyrus (left)	842	– 21	– 70	– 13	5.97	< 0.001
Precentral gyrus (left)	306	– 36	– 13	65	5.22	< 0.001
Middle temporal gyrus (left)	231	– 60	– 40	2	4.91	< 0.001
Middle temporal gyrus (right)	162	54	– 37	– 4	4.90	0.003
Inferior Frontal gyrus (right)	84	54	23	– 4	4.42	0.045
Contrast affect ambiguous vs. unambiguous						
Lingual gyrus (right)	369	15	– 82	– 7	4.92	< 0.001

Whole brain correction for task activation for WoF-task; *p*-value FWE corrected at cluster level

There were no significant differences at baseline between the two groups for any of the contrasts. Moreover, there were no significant differences between the groups in differences over time for any of the contrasts.

### Functional neuroimaging results—monetary incentive delay task

For the contrast anticipation reward > neutral we observed task activation in the left ACC. The contrast consumption reward > neutral at baseline showed more activation in the right supramarginal gyrus, right medial superior frontal gyrus extending to the ACC, bilateral insula, left inferior parietal gyrus, bilateral inferior frontal gyrus (IFG), the right middle cingulate gyrus, the left inferior temporal gyrus, and the right lingual gyrus. More activation was found for the contrast consumption loss > neutral at baseline in the right medial superior frontal gyrus, the right IFG, and the right inferior parietal gyrus. For the contrast consumption reward > loss at baseline there was more activation in the right postcentral gyrus and the right lingual gyrus.

More activation was found for the active treatment group compared to the sham group in the left cuneus and the left middle temporal gyrus at baseline for the contrast consumption reward > neutral (Table 3). There were no differences between the groups on the other contrasts.

**Table 3:** Functional neuroimaging results—monetary incentive delay task

(a) Task activation for MID-task						
Location	K	*x*	*y*	*z*	*t*-score	*p*-value
Contrast anticipation reward > neutral						
Anterior cingulate gyrus (left)	155	– 6	26	29	4.23	0.005
Contrast consumption reward > neutral						
Supramarginal gyrus (right)	571	48	– 40	44	7.53	< 0.001
Superior frontal medial gyrus (right)	655	6	29	44	7.48	< 0.001
Insula (right)	166	33	23	– 4	6.88	0.002
Inferior parietal (left)	404	– 54	– 40	47	6.39	< 0.001
Inferior frontal (right)	695	48	8	20	6.09	< 0.001
Middle cingulate gyrus (right)	216	0	– 22	32	5.90	0.001
Inferior temporal gyrus (left)	176	– 51	– 52	– 13	5.59	0.002
Lingual gyrus (right)	233	15	– 82	– 7	5.51	< 0.001
Inferior frontal gyrus (left)	301	– 39	5	29	5.25	< 0.001
Insula (left)	170	– 39	17	– 10	5.21	0.002
Contrast consumption loss vs. neutral						
Superior frontal medial gyrus (right)	129	9	29	41	5.72	0.010
Inferior frontal gyrus (right)	90	42	38	17	4.47	0.036
Inferior parietal gyrus (right)	181	45	– 43	47	4.35	0.002
Contrast consumption reward vs. loss						
Postcentral gyrus (right)	117	48	– 25	41	5.24	0.013
Lingual (right)	87	12	– 82	– 7	5.10	0.038

(b) MID-task, baseline, sham < active						
Location	*K*	*x*	*y*	*z*	*t*-score	*p*-value
Contrast consumption reward > neutral						
Cuneus (left)	1754	– 15	– 85	38	5.06	< 0.001
Middle Temporal gyrus (left)	198	– 63	– 25	2	4.22	0.001

(c) MID-task, group*time interaction, sham < active						
Location	*K*	*x*	*y*	*z*	*t*-score	*p*-value
Contrast consumption reward > neutral						
Inferior frontal (left)	185	– 48	26	11	4.61	0.003
Lingual & Middle temporal gyrus (right)	136	33	– 61	– 1	4.46	0.011

**Fig. 1:**
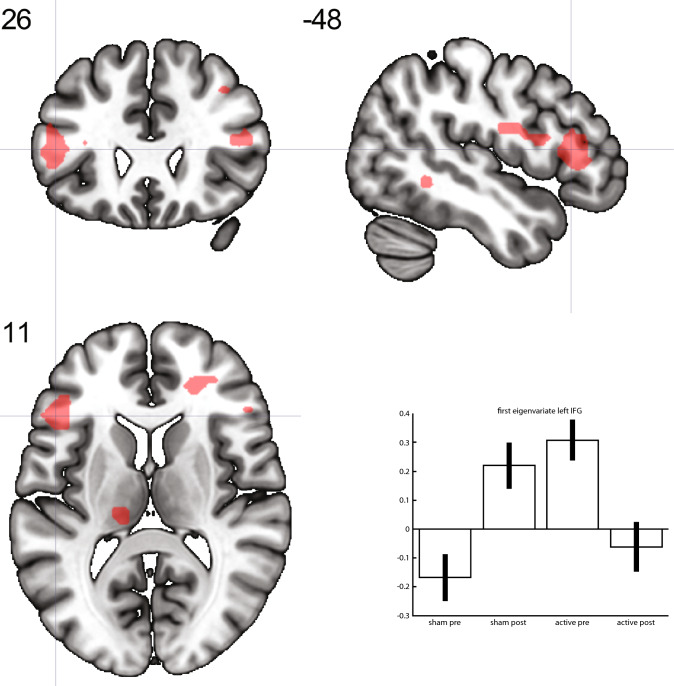
Larger decrease of activation in the active group compared to the sham group over time in the left IFG for the contrast consumption reward > neutral. Neurological convention

**Fig. 2:**
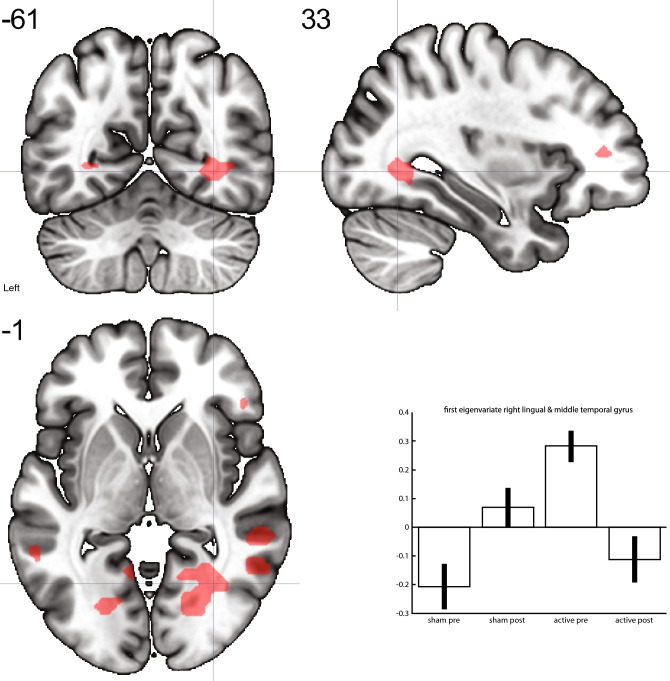
Larger decrease of activation in the active group compared to the sham group over time in a cluster comprising the right lingual gyrus and the posterior part of the middle temporal gyrus for the contrast consumption reward > neutral. Neurological convention

A group x time interaction was evident in the statistically significant differences between the groups in differences over time for the contrast consumption reward > neutral in the left IFG (*t*-score 4.61; *p* = 0.003) and in a cluster comprising the right lingual gyrus and the posterior part of the middle temporal gyrus (*t-*score 4.46; *p* = 0.011) (see Figs. 1 and 2; Table 3). Both clusters showed a larger decrease in activation in the active treatment group compared to the sham group. We found no relation between the change in depression severity as measured with the HAMD-17 and the average effect size of the interaction from the ROIs, i.e. left IFG and the posterior part of the middle temporal gyrus.

## Discussion

The aim of this study was to evaluate the effects of tPEMF on brain activation during emotion and reward processing. We showed that tPEMF stimulation decreased activation during reward-related processing in the left inferior frontal gyrus (IFG) and in a cluster comprising the right lingual gyrus and the posterior part of the middle temporal gyrus in MDD patients with a treatment-resistant depression. We did not find an effect of tPEMF on emotion processing during the WoF task. No behavioral effect or clinical effect [7] of tPEMF was found. These findings suggest that tPEMF may affect brain activation during reward processing in the absence of a clinical antidepressant effect.

Studies investigating the effects of global stimulation devices with tPEMF or comparable stimulation methods on brain activation are sparse. However, there is some evidence that tPEMF-like stimulation has an effect on glucose metabolism in the left IFG in healthy controls [31]. In this previous study the effect of the whole brain electromagnetic gradients of echo planar imaging (EPI), a standard for functional MRI, on brain glucose was investigated in 15 healthy male subjects, using Positron-Emission Tomography (PET) ^18^FDG imaging. All subjects underwent one MR-PET scan with EPI-gradients on, and one with EPI-gradient off (sham condition), the order of which was randomly assigned. When the EPI-gradient was turned on, a decrease in glucose metabolism in the left IFG was found in the absence of an effect on mood scores. This was also the case in clusters that included the inferior occipital, superior parietal and posterior insular cortices [31]. Our results are in line with this effect of global magnetic stimulation on the left IFG. A decrease in frontal activation during reward outcome has been reported before in healthy subjects in a test–retest design, albeit in a more ventral and medial region [32]. Thus, such a decrease in activation in a second measurement in a patient sample could perhaps be interpreted as "normalization", supporting a beneficial effect of tPEMF, whereas the lack of such a decrease may be associated with pathology. However, due to the lack of a healthy control group, there is no data to support this interpretation.

Consistent with the putative effect of tPEMF on reward-related brain activation, there is some evidence that tPEMF-like stimulation may have an effect on the growth of dopaminergic cells [21]. On the other hand, the left IFG and the extended area described as the ventrolateral prefrontal cortex (VLPFC), have both not been described as key areas of the reward network. Nevertheless, it could be suggested that they do play a role in associated regulatory processes [33]. The left IFG is implicated in cognitive and emotional control, especially cognitive reappraisal [34] and cognitive inhibition [35, 36]. Therefore, it could be speculated that during reward consumption the IFG inhibits ventral striatal regions subserving the subjective feeling of reward. Our finding of reduced IFG activation after tPEMF stimulation might indicate a reduction of this inhibitory effect and thereby an increase in the rewarding feeling. This explanation however is speculative and needs further confirmation. Still, it provides tentative indications of a possible effect of tPEMF on specific symptoms related to reward processing, for example anhedonia, more than on depressive symptoms in general. In that case, tPEMF might also have an effect on symptoms in other disorders where impairments of reward processing play a role, like schizophrenia and addiction. Therefore, our finding of change in reward-related brain activation may also be relevant for future research to tPEMF as a novel treatment option for psychiatric symptoms in other disorders than MDD.

Apart from the effect on the left IFG, we found a group-by-time interaction indicating decreased activation during reward consumption in a cluster comprising the right lingual gyrus and the posterior part of the middle temporal gyrus. Interestingly, MDD has been associated with more activation in the cuneus and lingual gyrus during reward processing [19]. The observed decrease over time in the active treatment group might suggest the normalization of brain activation patterns in the lingual gyrus as a result of tPEMF treatment. However, due to the lack of a healthy control group this could not be tested, so such an interpretation is tentative at best. Surprisingly, we also observed higher activation on baseline in the active treatment group compared to the sham group in this cluster. This might also suggest that the effect is related to regression to the mean. Therefore, at this point, it is difficult to draw definitive conclusions.

Apart from the effects on reward processing, we also studied the effects of tPEMF on emotion processing. For this, we used the WoF task, an emotional attention task that probes neural circuitry underlying affective appraisal of multiple simultaneously presented faces [13]. This task was used earlier in healthy controls [13], anxious individuals [37, 38] and patients with schizophrenia [39], but not yet in MDD. In healthy controls, Simmons and colleagues reported activation of the dorsal ACC, dorsolateral PFC, and posterior parietal cortex during ambiguous trials in general [13]. During emotionally ambiguous trials, more activation was found in the right supramarginal gyrus, right superior temporal gyrus, and the ventromedial PFC (including the ventral ACC) compared with ambiguous gender trials [13]. We observed task activation during ambiguous affective trials compared to ambiguous gender trials in the left lingual gyrus, left precentral gyrus, bilateral MTG and the right IFG, and during ambiguous affective trials compared to unambiguous emotional trials more activation in the right lingual gyrus. Thus, even though there was little overlap between the areas found by Simmons et al., we found involvement of areas known to subserve processing of faces and emotional expressions (40). The absence of overlap could be partly due to the difference in population and it might be that patients with MDD show a different pattern of activation during this task.

Brain areas involved in emotional processing, like the dorsal ACC and ventromedial PFC, as well as the amygdala and parahippocampal cortex [14] can be indirectly influenced after left DLPFC rTMS stimulation due to the high connectivity with superficial cortical areas [16, 41]. We did not find an effect of tPEMF on these brain areas or an effect of tPEMF on behavioral emotional processing data, suggesting that tPEMF with our settings has no impact on these deeper areas during emotional processing.

### Limitations

The current study focused on the differences between tPEMF-stimulation and sham treatment in a randomized clinical trial. As we did not have a healthy control group it was not possible to draw firm conclusions regarding the level of task-related activation during the ambiguous emotional faces task. In addition, because tPEMF did not show clinical improvement [7], we could not relate our fMRI findings to clinical outcomes. On the other hand, this implies that changes on the neural level could be more sensitive to change as we did find differences in brain activation related to tPEMF stimulation.

## Conclusion

We evaluated the effects of tPEMF on brain activation by investigating different processes underlying hallmark symptoms of MDD: emotional and reward processing. We found a decrease in brain activation in the left IFG during reward-outcome processing as a result of tPEMF-stimulation, while no effect of tPEMF on emotional processing was found. These findings suggest that tPEMF may affect relevant brain activation (though in our study in the absence of a clinical antidepressant effect) and encourage further investigation with different parameters (e.g., regarding intensity, duration and location of stimulation).

## Data Availability

Data is available upon request.
